# Phylogenetic Analysis and Genome-Wide Association Study Applied to an Italian *Listeria monocytogenes* Outbreak

**DOI:** 10.3389/fmicb.2021.750065

**Published:** 2021-11-04

**Authors:** Alexandra Chiaverini, Fabrizia Guidi, Marina Torresi, Vicdalia Aniela Acciari, Gabriella Centorotola, Alessandra Cornacchia, Patrizia Centorame, Cristina Marfoglia, Giuliana Blasi, Marco Di Domenico, Giacomo Migliorati, Sophie Roussel, Francesco Pomilio, Yann Sevellec

**Affiliations:** ^1^National Reference Laboratory for Listeria monocytogenes, Istituto Zooprofilattico Sperimentale dell’Abruzzo e del Molise G. Caporale, Teramo, Italy; ^2^Istituto Zooprofilattico Sperimentale dell’Umbria e delle Marche “Togo Rosati”, Perugia, Italy; ^3^National Reference Centre for Whole Genome Sequencing of Microbial Pathogens Database and Bioinformatic Analysis, Istituto Zooprofilattico Sperimentale dell’Abruzzo e del Molise G. Caporale, Teramo, Italy; ^4^Laboratoire de Sécurité des Aliments, Agence Nationale de Sécurité Sanitaire de l’Alimentation, de l’Environnement et du Travail, Université PARIS-EST, Maisons-Alfort, France

**Keywords:** *Listeria monocytogenes*, GWAS, outbreak, WGS, ST7

## Abstract

From May 2015 to March 2016, a severe outbreak due to *Listeria monocytogenes* ST7 strain occurred in Central Italy and caused 24 confirmed clinical cases. The epidemic strain was deeply investigated using whole-genome sequencing (WGS) analysis. In the interested area, the foodborne outbreak investigation identified a meat food-producing plant contaminated by the outbreak strain, carried by pork-ready-to-eat products. In the same region, in March 2018, the epidemic strain reemerged causing one listeriosis case in a 10-month-old child. The aim of this study was to investigate the phylogeny of the epidemic and reemergent strains over time and to compare them with a closer ST7 clone, detected during the outbreak and with different pulsed-field gel electrophoresis (PFGE) profiles, in order to identify genomic features linked to the persistence and the reemergence of the outbreak. An approach combining phylogenetic analysis and genome-wide association study (GWAS) revealed that the epidemic and reemergent clones were genetically closer to the ST7 clone with different PFGE profiles and strictly associated with the pork production chain. The repeated detection of both clones was probably correlated with (i) the presence of truly persistent clones and the repeated introduction of new ones and (ii) the contribution of prophage genes in promoting the persistence of the epidemic clones. Despite that no significant genomic differences were detected between the outbreak and the reemergent strain, the two related clones detected during the outbreak can be differentiated by transcriptional factor and phage genes associated with the phage LP-114.

## Introduction

Invasive listeriosis is a severe foodborne disease caused by *Listeria monocytogenes* (*Lm*) and characterized by high hospitalization and fatality rates. Invasive infections mainly occur among pregnant women, the elderly, and immunocompromised people causing septicemia, meningitis, and maternal–neonatal infections.

In Europe, in the framework of 2019 data collection, 28 Member States reported 2,621 confirmed invasive cases of listeriosis with an overall case fatality of 17.6%. The figures are increasing compared to 2018 and 2017 (European Food Safety Authority and European Centre for Disease Prevention and Control, 2021).

Clinical listeriosis cases can occur as outbreaks (Gómez-Laguna et al., 2020; Lepe, 2020), due also to the increasing globalization of the food trade (EFSA Panel on Biological Hazards, Koutsoumanis et al., 2019). The largest European outbreak of listeriosis during the 25 past years, which included more than 130 laboratory-confirmed cases, was described in Germany during the period 2018–2019 (Halbedel et al., 2020).

To date, whole-genome sequencing (WGS) provides the highest possible subtyping resolution improving the surveillance, outbreak investigation, source attribution, and microbial population studies of *Lm* (Moura et al., 2016; Portmann et al., 2018; Allard et al., 2019; Hurley et al., 2019; Pietzka et al., 2019).

Moreover, as reported in the European Food Safety Authority and European Centre for Disease Prevention and Control (2021) (EFSA) report, high-risk pathogenic *Lm* strains can be also identified based on the presence of phenotypic properties, such as virulence potential, but also persistence, or growth/survival under stress conditions prevailing in food or the host, using comparative genomics studies or genome-wide association studies (GWAS). This latter approach compares a large set of genomic data and associates them to specific phenotypic traits, allowing for the identification of markers or indicators of specific phenotypes. The GWAS analysis has been successfully used for identifying *Lm* genetic determinants involved in biofilm formation (Lee et al., 2019), persistence (Fritsch et al., 2019; Palma et al., 2020), and virulence (Sévellec et al., 2020).

From May 2015 to March 2016, a severe outbreak due to *Lm* ST7 strains occurred in Central Italy (Marche Region) and involved 24 confirmed clinical cases. The epidemic strain had an uncommon pulsed-field gel electrophoresis (PFGE) profile (GX6A16.0183, GX6A12.0063), showing a close genetic relationship with strains characterized by a different PFGE profile (GX6A16.0060, GX6A12.0063) and detected in three clinical cases that occurred in 2014, 2015, and 2016, respectively. During the outbreak investigation, several environmental and food samples were collected and analyzed to detect *Lm* and in particular the *Lm* epidemic strain.

A hog head cheese collected in a retail store (RS1) tested positive for the epidemic strain leading to the identification of the pork meat food-processing plant (FPP1) producing it, as the main source of the epidemic strain (Duranti et al., 2018). Starting from the FPP1, through trace-back investigation the epidemic cluster was detected from foods and environmental samples of one of the FPP1’s suppliers. Moreover, during the trace-forward investigation the epidemic strain was isolated in one plant that repackaged the FPP1’s products and in different retailers receiving products from the FPP1 (Duranti et al., 2018; Supplementary Table 1).

The epidemic strain reemerged in March 2018 in the same Region causing a new sporadic case of listeriosis in a 10-month-old child (Orsini et al., 2018b). Very interestingly, during the epidemiological investigation, from the environmental samples collected in the child’s home, both the PFGE profiles (the epidemic strain and the one closely related to it) were isolated. Moreover, the reemerging epidemic strain was also isolated from the environment of an RS traced through the epidemiological questionnaire.

Although WGS has allowed the source attribution and the resolution of the outbreak, there are no clear aspects on the evolutionary dynamics behind the coexistence of two related strains during the outbreak and on their reemergence after 2 years.

The aim of this study was to investigate the phylogeny of the epidemic and reemergent strains over time and to compare them with the closer clone with different PFGE profiles. We used a combined approach including phylogeny analysis and GWAS to compare all the interested genomes in order to identify genomic features potentially linked to the persistence and the reemergence of the outbreak. The data were retrieved from the database cured by the Italian National Reference Laboratory for *Lm* (ItNRL *Lm*) and the National Reference Centre for Whole Genome Sequencing of microbial pathogens: database and bioinformatic analysis.

## Materials and Methods

### Dataset Collection

In this study, we tested 186 strains, including 178 *Lm* strains collected during the outbreak (2015–2016) (n. 144) (Duranti et al., 2018) and the reemergence in 2018 (n. 34) (Orsini et al., 2018a). Among them, 32 were isolated from clinical cases and 146 were from food or FPP environment.

Additionally, eight ST7 strains not correlated with the outbreak were randomly selected as “Outgroup” (Og) from the ItNRL *Lm* database as well. Metadata of the entire dataset are summarized in Supplementary Table 1.

In the dataset, 174 of 178 strains showed the PFGE patterns GX6A16.0183 and GX6A12.0063 linked to the outbreak (including “Epidemic Cluster—EpiCl” and “Re-emergence Cluster—ReE” of 2018); four strains showed different PFGE combined profiles (GX6A16.0060, GX6A12.0063) (“Cluster 2”—C2) detected in 2015–2016 and during the reemerging outbreak (ReE) in 2018 (2018.TE.6199.1.14).

To be included in the outbreak, the 178 strains were screened with a real-time PCR screening assay performed and described previously by Torresi et al. (2020).

### Next-Generation Sequencing and Data Analysis

DNA of all the 186 *Lm* strains was extracted using the Maxwell 16 Tissue DNA Purification Kit (Promega Italia Srl, Milan, Italy) according to the manufacturer’s protocol, and the purity of the extracts was evaluated by NanoDrop (Thermo Fisher Scientific, Waltham, MA, United States). Starting from 1 ng of input DNA, we used the Nextera XT DNA chemistry (Illumina, San Diego, CA, United States) for library preparation according to the manufacturer’s protocols. WGS was performed on the NextSeq 500 platform (Illumina, San Diego, CA, United States) with the NextSeq 500/550 Mid Output Reagent Cartridge v2 (300 cycles, standard 150-bp paired-end reads).

For the analysis of WGS data, an in-house pipeline was used (Cito et al., 2018) which included steps for trimming (Trimmomatic v0.36) (base quality parameters—leading: 25; trailing: 25; sliding window: 20:25) (Bolger et al., 2014) and quality control check of the reads (FastQC v.0.11.5) (Wingett and Andrews, 2018). Genome *de novo* assembly of paired-end reads was performed using SPAdes v3.11.1 (Bankevich et al., 2012) with default parameters (–only-assembler –careful –k21, 33, 55, 77). Then, the genome assembly quality check was performed with QUAST v.4.3 (Gurevich et al., 2013). All the genomes that met the quality parameters recommended by Timme et al. (2020) were used for the subsequent analysis steps.

Sequence type (ST) and the clonal complex (CC) were deducted *in silico* using the related function available on the BIGSdb-Lm database.^1^

### cgMLST and Single-Nucleotide Polymorphism Analysis

For verifying the relatedness among the isolates from the outbreak and those belonging to ST7 and collected during the 2000–2020 period, a core-genome MLST (cgMLST) analysis was performed. As allele-calling method, a chewBBACA pipeline (Silva et al., 2018) was executed according to the Pasteur Institute scheme using the 1,748 loci (Moura et al., 2016). The software GrapeTree (Zhou et al., 2018) was performed for the visualization of the Minimum Spanning tree (MSTreeV2).

The single-nucleotide polymorphism (SNP) analysis was conducted using the CFSAN pipeline (Davis et al., 2015), and the sequence reads were mapped against an EGD-e reference genome (NC_003210.1). The resulting maximum likelihood (ML) tree was visualized using Interactive Tree of Life (iTOL)^2^ and then utilized for the following pan genome analysis.

### Pangenome-Wide Association Study

The genome annotation of 186 *Lm* strains was carried out using Prokka (Seemann, 2014) with default parameters. Using the assemblies as an input Prokka produced GFF files, including sequences and annotations, which were used to extract the pangenome with Panaroo (Tonkin-Hill et al., 2020). For the visualization of the Pangenome based on gene presence/absence, Phandango was used (Hadfield et al., 2018). The genes were considered present or absent based on Panaroo prediction using a 98% identity cutoff after annotation correction and CDS realignment. The pan genome prediction was corrected using the ML tree generated using the CFSAN pipeline.

Then, the pangenome-wide association study (pan-GWAS) was used to identify genes significantly associated with the EpiCl, ReE, and C2 strains. GWAS was performed using Pyseer (Lees et al., 2018) which assessed the presence/absence patterns of genes in three subpopulations [“Epidemic Cluster + Re-emergence (EpiCl + ReE),” “Cluster2 (C2),” and “Re-emergence (ReE)”] based on linear mixed model (LMM). The genes with significant association were reported for verifying any differences between EpiCl + ReE, ReE, and C2.

In order to check the confounding population stratification, a pairwise comparison was performed based on the ML tree for population structure correction. Moreover, information regarding the obtained significant genes was retrieved using BLAST.^3^

### Virulence, Persistence, and Antimicrobial Gene Identification

In order to detect the absence/presence of loci encoding virulence- and persistence-associated genes, all the 186 *Lm* genomes were manually screened using the tools “Virulence,” “Antibiotic Resistance,” “Metal and Disinfectants Resistance,” “Stress Islands,” and “Listeria Genomic Islands,” available on the BIGSdb platform.^4^

Furthermore, PlasmidFinder (Carattoli et al., 2014) was queried through the software ABRicate v.0.8 (Seeman, 2014) in order to evaluate the presence of mobile elements and to verify if they carried resistance genes.

## Results

### Whole-Genome Sequencing Analysis

For the 186 genomes analyzed, we obtained sequence data according with the quality control thresholds recommended for *Lm* as average read quality ≥30, average coverage ≥ 20, *de novo* assembly seq. length between 2.7 and 3.2 Mbp, and number of contigs ≤300 (Supplementary Table 2). Furthermore, the MLST analysis confirmed that all the 186 genomes belonged to ST7 and CC7.

### Phylogenetic Analysis

**FIGURE 1:**
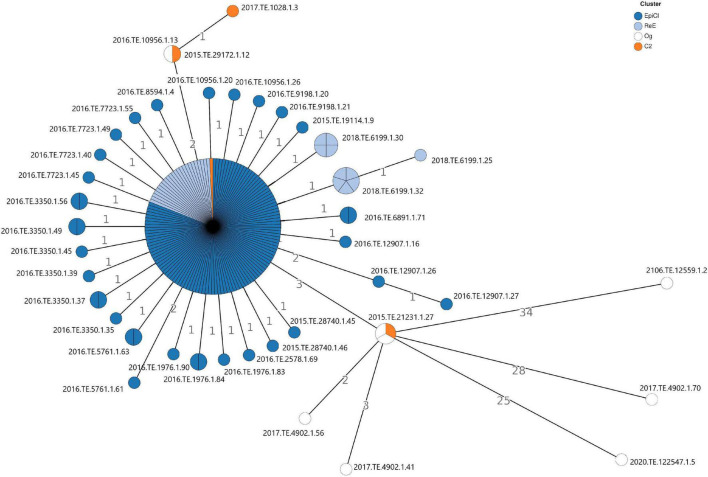
Cluster analysis based on gene-by-gene results of the 186 *L. monocytogenes* isolates tested in this study. The nodes are colored according to the cluster (Epidemic, Cluster2, and Re-emergence Cluster). The number reported in the branches indicate the allelic differences existing between the isolates.

The cgMLST analysis showed that no significant allelic distance was highlighted between EpiCl, C2, and ReE strains. Indeed, most of the *Lm* EpiCl strains (n. 106), ReE strains (24 genomes), and one C2 strain (2018.TE.6199.1.14) shared the same allelic profile (Figure 1). Regarding the remaining strains belonging to the EpiCl, those isolates showed to be distant one to two alleles from the main group. Additionally, the same trend was followed by the 10 isolates of the ReE not included in the abovementioned clone. The remaining three strains belonging to C2 showed a closer relation with some Og strains. In fact, two strains (2015.TE.29172.1.12 and 2017.TE.1028.1.3) showed to be distant 0–1 alleles from 2016.TE.10956.1.13; meanwhile, the clinical strain (2015.TE.21231.1.27) matched with two outgroup strains detected in 2019.

**FIGURE 2:**
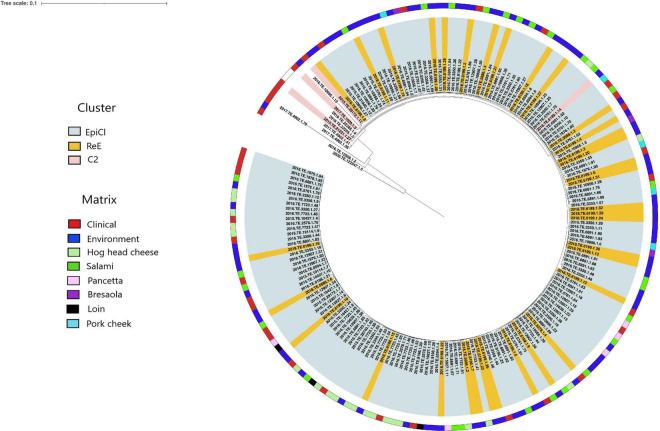
Maximum likelihood (ML) tree obtained from the single-nucleotide polymorphism (SNP) analysis. The ranges are colored according with the cluster, and the first layer represents the source of isolation as shown in the legend. The ML tree was midpoint rooted.

The results obtained from the SNP analysis showed to be congruent with them of cgMLST analysis. The “ML tree” obtained showed that the first cluster included 140 EpiCl strains from the 2015 to 2016 and 34 ReE strains from 2018 (Figure 2). The ML tree showed that the EpiCl, including the ReE, shared a common ancestor with the C2 cluster, which was distant an average of 28 SNPs (min 0, max 80) from the outgroup strains.

According to the SNP matrix (Supplementary Table 3), the SNP distances detected within the EpiCl, ReE, and C2 clusters were 3 (min 0, max 10), 1 (min 0, max 5), and 9 (min 4, max 14), respectively.

The ReE strains were spread out with three SNPs on average (min. 0, max. 10) from EpiCl strains. Moreover, the clinical strain isolated from the child (2018.TE.5376.1.4) in 2018 was distant two and three SNPs from two EpiCl strains detected from the FPP environment (2016.TE.10956.1.29) and salami (2016.TE.6891.1.86), respectively. Additionally, the SNP matrix showed that the ReE strain 2018.TE.5376.1.4 was distant one SNP from the clinical EpiCl strain 2016.TE.6891.1.6.

Furthermore, the SNP analysis showed a close correlation between some EpiCl clinical strains and those isolated from the environment during the ReE of the outbreak. In particular, seven clinical isolates (2015.TE.28740.1.46, 2016.TE.5761.1.61, 2016.TE.5761.1.62, 2016.TE.5761.1.66, 2016.TE.1976.1.90, 2016.TE.1976.1.82, and 2016.TE.5761.1.63) were spread out with three SNPs on average (min 0, max 6). Meanwhile, 17 ReE isolates from environment (2018.TE.6199.1.5, 2018.TE.6199.1.7, 2018.TE.6199.1.12, 2018.TE.5686.1.2, 2018.TE.6199.1.15, 2018.TE.5686.1.6, 2018.TE.6199.1.9, 2018.TE.6199.1.8, 2018.TE.6199.1.31, 2018.TE.6199.1.13, 2018.TE.5686.1.4, 2018.TE.5686.1.3, 2018.TE.6199.1.4, 2018.TE.6199.1.20, 2018.TE.6199.1.3, and 2018.TE.6199.1.6) were distant three SNPs on average (min 0, max 4) from EpiCl strains isolated from hog head cheese, salami, pancetta, loin, and bresaola.

Five outgroup strains clustered together with those belonging to C2, except for 2018.TE.6199.1.14. Meanwhile, the remaining three strains (2020.TE.122547.1.5, 2017.TE.4902.1.70, and 2016.TE.12559.1.2) showed to be phylogenetically distant from the genomes of the dataset.

Finally, the C2 strain detected during the ReE (2018.TE.6199.1.14) showed to be distant three SNPs from EpiCl strains isolated from hog head cheese, salami, pork cheek, and environment.

### Genome-Wide Association Study

**FIGURE 3:**
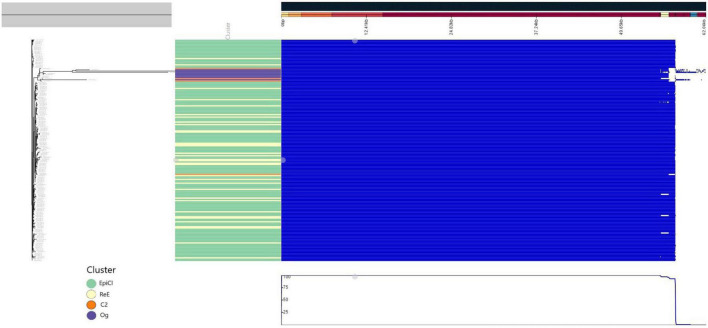
Pangenome representation of all 186 *L. monocytogenes* isolates used in this study. The genes were considered present (blue color) or absent (white) based on Panaroo prediction using a 98% identity cutoff after annotation correction and coding sequence (CDS) realignment. The pan genome prediction was corrected using the maximum likelihood (ML) tree generated using the CFSAN pipeline. The metadata were colored in “Cluster” part according to the cluster (green for “Epi-Cl,” orange for “C2,” yellow for “Re-E,” and purple for “Og”).

A predicted pan genome of 190 *Lm* strains was created using annotated draft assemblies. We identified a core of 2,768 coding sequences (CDSs) present in each of the sequenced isolates, while the total accessory genome was composed of an additional 333 CDSs. Identified genes included 62 soft-core genes (i.e., in more than 95% of the genomes), 46 shell-genes present in 15–95% of the genomes, and 225 genes present in <15% of the genomes. According with the phylogenetic analysis, the pan genome of all the isolates belonging to the EpiCl, including those from the ReE, showed to be very similar, as reported in Figure 3; meanwhile C2 and Og strains showed some difference and deletions.

Based on the results of the LMM, significance thresholds of 2.30 (C2) and 2.74 (EpiCl + ReE) were calculated. The *p*-values below these thresholds were deemed as significantly associated with the specific subpopulation (Supplementary Table 4). Meanwhile, no significant genes associated with only the ReE subpopulations were detected.

Genome-wide association study revealed that the EpiCl + ReE and C2 subpopulations were characterized by the presence of genes encoding holin, class I SAM-dependent methyltransferase, single-stranded DNA-binding protein, pentapeptide repeat-containing protein, sugar-phosphate nucleotidyltransferase, site-specific integrase, and N-acetylmuramoyl-L-alanine amidase. Phage terminase small subunit P27 family, phage tail protein, and three hypothetical proteins, one of which was harbored by Listeria phage LP HM00113468, were also detected (Supplementary Table 4).

Furthermore, several proteins phage-related with several functions such as DUF1642 domain-containing protein, which is harbored in Listeria phage LP-114 (NC_024392.1), phage gp6-like head-tail connector, HK97 gp10 family phage, HK97 family phage prohead protease, phage tail tape measure, phage major capsid and phage portal proteins, and 13 more hypothetical proteins, which have different functions, were significantly associated with C2 strains (Supplementary Table 4).

### Virulence, Persistence, and Antimicrobial Gene Identification Results

No significant differences were highlighted among the EpiCl, C2, ReE, and Og strain genome (Supplementary Table 5).

Regarding virulence genes, 57 different genes, including incomplete alleles, were detected in overall 186 *Lm* strains used in this study. Moreover, two epidemic strains isolated from salami (2016.TE.5761.1.69 and 2016.TE.6891.1.72) carried also incomplete alleles in LM9005581_70010, LM9005581_70011, and LM9005581_70012 genes. No isolates showed to carry a PMSC mutation in the *inlA* gene encoding for a truncated internalin A protein. Moreover, in all the genomes tested *prfA*, *plcA*, *hly*, *mpl*, *actA*, and *plcB*; the adherence genes *ami* and *lapB*; and the *comK* prophage were found. None of the strains carried LIPI3 island, *gltA*, and *gltB* genes.

Six Listeria genomic islands were detected in 186 strains while LGI-2_LMOSA2269, LGI-2_LMOSA2270, LGI-2_LMOSA2271, and LGI-2_LMOSA2272 were missing in four clinical strains belonging to EpiCl and C2. Finally, 2016.TE.5761.1.69 and 2016.TE.6891.1.72 carried incomplete alleles in LGI-2_LMOSA2270.

Using the functions “Metal and Disinfectants Resistance,” no disinfectant resistance genes and toxic compounds were detected except for the Og strain 2020.TE.122547.1.5 (Tn6188_qac). Meanwhile, the Stress-Survival Islet 1 (SSI-1) for tolerance to environmental stresses was identified in the 186 genomes of the dataset.

The 186 *Lm* genomes showed to carry lincomycin- (*lmo0919*) and sulfonamide- (*sul*) intrinsic resistance genes; meanwhile, the epidemic cluster 2020.TE.3350.1.8 isolated from hog head cheese harbored also tetracycline resistance genes *tetM* and *tetS.*

The replicon plasmid J1776 (CP006612) was detected in all the isolates belonging to EpiCl, ReE, and C2. This plasmid harbored a plasmid-borne cadmium resistance gene contained in transposon Tn522.

## Discussion

In this study, we explored the genome of two ST7 clones related to a severe *Lm* outbreak that occurred in Central Italy between 2015 and 2016 and reemerged in 2018. The main aim was to identify any genetic determinant involved in the coexistence of the two related clones during the outbreak and in the reemergence using different WGS approaches such as cluster analysis and GWAS. The cluster analysis, based on cgMLST and SNP analysis, was performed to define the genetic relationships between EpiCl strains and C2 and ReE strains.

Despite that the allele-based method did not reveal any significant difference among the isolates related to the outbreak, the results obtained were congruent with SNP analysis showing that most of the *Lm* EpiCl isolates and the ReE shared the same allelic profile. Interestingly, one strain with a PFGE profile matching with C2, detected in 2018 during the reemergence, showed the same allelic profile of EpiCl and ReE strains.

The SNP analysis confirmed the allelic close correlation between EpiCl and ReE strains. More in detail, the cluster analysis revealed the strict correlation between the ReE clinical strains isolated from a 10-month-old child and two strains isolated from an environmental swab and a salami sample isolated during the outbreak. Moreover, the same correlation was highlighted between clinical strains, environmental strains, and food strains (hog head cheese, salami, pancetta, loin, and bresaola) detected during EpiCl and ReE.

In a recent study, Scaltriti et al. (2020) stated that the Marche outbreak originated from a cluster coming from a neighbor Region (Emilia-Romagna), whose source of contamination was still unknown.

To date, the source for the clinical ReE strain has not yet been attributed. Phylogenetic analytical results showed the key role of the pork production chain (hog head cheese, salami, and pancetta) in keeping the epidemic clone in circulation, or the possible reintroduction along the food production chain. Those hypotheses could be supported by the pairwise distances detected among EpiCl and ReE strains.

The results suggested the presence of a persistent clone over time, probably resident in a specific FPP environment and the possible reintroduction of closely related clones along the entire food chain, as already described by Knudsen et al. (2017).

Moreover, the closer similarity existing between some ReE strains and the EpiCl clones isolated from a FPP that was promptly closed in 2016 suggested the presence of additional contaminated FPPs downstream in the supply chain, as suggested by Duranti et al. (2018). Those FPPs were probably represented by retail shop environments where the epidemic strain was introduced during the outbreak and where they harbored and persisted.

The hypothesis is supported by Stasiewicz et al. (2015) and Palma et al. (2020), who affirmed the possible presence of identical or nearly identical strains in different retail environments with subsequent persistence in some of them.

Furthermore, the possible existence of a common ancestor between the two similar clones detected during the outbreak (EpiCl and C2) could be confirmed by the close correlation existing between the only isolate belonging to C2 detected during the reemergence and EpiCl strain isolates from hog head cheese, salami, and pork cheek.

Maury et al. (2016) reported that CC7 strains, unlike well-adapted FFP environmental CC9 and C121 strains, are rarely isolated from food and not frequently associated with clinical infections. Therefore, this clone was not recognized as hypervirulent as CC1, CC2, CC4, and CC6.

Indeed, according with the definition of hypervirulent clones assessed by the previous mentioned authors, our results showed that no *Listeria* pathogenicity island 3 (LIPI-3), encoding the hemolytic and cytotoxic factor Listeriolysin S (LLS) (Tavares et al., 2020), was detected in our dataset. In contrast, all the strains carried a full-length *InlA* gene and *gtcA*, responsible for teichoic acid biosynthesis (Faith et al., 2009). Consistent with their belonging to serotype 1/2a, the strains lacked the genes *gltA* and *gltB*, which are serotype 4b-specific genes (Lei et al., 2001; Raschle et al., 2021).

Moreover, investigating the virulence profiles, no significant differences were detected among the different clusters (EpiCl, C2, ReE, and Og). However, it was evident that all the 190 isolates harbored *comK* prophage, LIPI2_inlII, and LIPI1, which consists of six virulence genes (*prfA*, *plcA*, *hly*, *mpl*, *actA*, and *plcB*). This prophage is known to play a key role in the host infection (Moura et al., 2016).

Regarding the genomic features involved in persistence, no efflux-pump genetic determinants were identified. Only one strain belonging to the Og cluster showed to harbor the transposon-borne *qac* gene, encoding an efflux-pump specific for quaternary ammonium compounds and in particular associated with the export of benzalkonium chloride (Maury et al., 2019; Guidi et al., 2021).

The strains in this dataset presented the SS1 islet which contributes to the growth of *Lm* at low pH, high salt concentrations, and refrigeration temperature (Palma et al., 2020). It was previously correlated with biofilm formation in the FPP environment (Guidi et al., 2021). The SS2 islet was absent in all our collections except for one strain that presented the lin0464 gene. Those results contrasted with a previous study by Palaiodimou et al. (2021) that demonstrated that food-persistent isolates usually present both SSI.

Our results showed that Listeria Genomic island 2 (LGI2), LGI-2_LMOSA2310 homolog, and LGI3 (LGI-3_LmUB3PA_1685) were found in the 186 genomes analyzed. LGI2 seemed not to carry arsenic resistance genes, unlike the homolog described by Lee et al. (2017) in serotype 4b strains. Meanwhile, LGI3 is a novel genomic island first described in CC101 isolates by Palma et al. (2020), which includes cadmium resistance determinants (*cadA1C*) and transposase *Tn3* and can contribute to the adaptation of *Lm* in the FPP environment.

Cadmium transporter and transposase *Tn552* were detected, carried by the plasmid J1776, which was common to all the isolates belonging to the EpiCl, C2, and ReE strains. This plasmid was usually found in CC9 and CC121 considered as clones persistent in FPPs (Guidi et al., 2021).

GWAS analysis confirmed that EpiCl and ReE strains do not have significant differences in their gene content; meanwhile, the C2 strains were successfully discriminated from EpiCl + ReE through a prophage-associated cluster of 11 genes with different functions. Specifically, C2 strains showed a high association with genes encoding the DUF1642 domain-containing protein, which is harbored in Listeria phage LP-114 (NC_024392.1) as described by Denes et al. (2014), phage gp6-like head-tail connector, HK97 gp10 family phage, HK97 family phage prohead protease, phage tail tape measure, phage major capsid, and phage portal proteins. In bibliography, several authors (Orsi et al., 2008; Knudsen et al., 2017; Pirone-Davies et al., 2018; Palma et al., 2020) highlighted the link between prophage and *L. monocytogens*’ fitness to specific niches in the FPPs. Suzuki et al. (2020), instead, showed that the presence of a lysogenic phage in a genome can provide protection against similar phages.

Furthermore, the XRE family transcriptional regulator, which is involved in stress tolerance and virulence (Hu et al., 2019), enriched the C2 strains, as the MerR family transcriptional regulator.

Indeed, the MerR family is a group of transcriptional activators that respond to environmental pressure factors as oxidative stress, heavy metals, and antibiotics (Brown et al., 2003) and is involved in biofilm formation (Huang et al., 2012). Moreover, among the transcriptional factors, genes encoding the helix–turn–helix domain-containing protein, which is involved in DNA repair and replication (Aravind et al., 2005), and RNA polymerase subunit sigma were associated with C2. In particular, the RNA polymerase subunit sigma has a main role for activating gene expression under normal growth or physiological changes such as *prfA*, *qoxABCD* (which encodes enzyme for oxidative stress response), and *cggR*, which encodes the central glycolytic gene regulator (Boonmee et al., 2019).

The genes associated with both EpiCl + ReE strains and C2 have different functions. Holin, a phage protein encoded by a gene adjacent to the endolysin gene, contains one transmembrane α-helical sequence that is essential for bacterial cell lysis during bacteriophage infection (Song et al., 2020). In addition to this phage protein, phage terminase small subunit P27 family, phage tail protein, and one hypothetical protein harbored in Listeria phage LP HM00113468 (NC_049900.1) enriched those clusters. These findings were in accordance with those obtained by Palma et al. (2020), who highlighted the impact of mobile genomic elements as phage on the genome evolution of *Lm* and the subsequent adaptation of persistent clones in the FPP environment.

Moreover, the GWAS results revealed the association of the EpiCl + ReE and C2 clusters with cassette genes including genes that encode single-stranded DNA-binding protein, site-specific integrase, and hypothetical protein that was annotated in the ReE clinical strain previously published by Orsini et al. (2018b) (CP029372.1).

Finally, also the class I SAM-dependent methyltransferase, which regulates a wide range of biological processes through the modification of biological macromolecules such as DNA, RNA, polysaccharides, and lipids (Currie et al., 2017), enriched those clusters.

## Conclusion

In this study, genomes of EpiCl and ReE *Lm* strains, which caused a severe listeriosis outbreak, occurred in Central Italy and a reemergence after 2 years was investigated vs. a similar clone (C2) and Og strains, in order to identify the genetic determinants possibly involved in the outbreak and in the reemergence of the epidemic strain.

The cgMLST analysis was not enough discriminatory to identify differences; on the contrary, the SNP analysis provided significant elements that could explain the dynamics of the reemergence. The phylogenetic analysis uncovered the different evolution path of the clones EpiCl and C2, characterized by a common ancestor.

Moreover, the repeated detection of both clones suggested the strict correlation among EpiCl and C2 clones, together with ReE, all strictly linked to the pork production chain, probably correlated with the presence of truly persistent clones and the repeated introduction of new ones.

In addition, the GWAS results, together with the genomic characterization, revealed the contribution of prophage genes and transcriptional factors in promoting the persistence of EpiCl, C2, and ReE clones in the FPP environment and as a consequence in finished products.

The combined approach (phylogenetic analysis and GWAS) could be applied in genomic datasets including a larger number of isolates in order to correlate them and to unveil the evolutionary pathway of ST7 strains. This combined approach could help to understand if strains causing outbreak are still circulating in order to plan and carry out a more deep investigation in FPP environments and clean the environment, avoiding a possible reemergence of *Lm* in the future.

## Data Availability Statement

The datasets presented in this study can be found in online repositories. The names of the repository/repositories and accession number(s) can be found in the article/Supplementary Material.

## Author Contributions

ACh and YS conceptualized and designed the study. ACh performed the bioinformatics analysis. FG, GC, MT, VA, ACo, MD, PC, and CM performed all the microbiological and molecular analyses and contributed to the interpretation of data. ACh, FG, and YS wrote the original draft. YS, SR, GM, GB, and FP supervised the work and contributed to the interpretation of data. All the authors contributed to the manuscript drafting.

## Conflict of Interest

The authors declare that the research was conducted in the absence of any commercial or financial relationships that could be construed as a potential conflict of interest.

## Publisher’s Note

All claims expressed in this article are solely those of the authors and do not necessarily represent those of their affiliated organizations, or those of the publisher, the editors and the reviewers. Any product that may be evaluated in this article, or claim that may be made by its manufacturer, is not guaranteed or endorsed by the publisher.

## Footnotes

1
http://bigsdb.pasteur.fr/listeria

2
https://itol.embl.de/

3
https://blast.ncbi.nlm.nih.gov/Blast.cgi

4
https://bigsdb.pasteur.fr/

## Supplementary Material

Supplementary Table 1 Metadata of all 186 *L. monocytogenes* genomes used in this study. In column “Cluster” are reported the following acronyms: “EpiCl” (Epidemic Cluster), “Re-E” (Re-emergence Cluster), and C2 (Cluster 2).

Supplementary Table 2 QC data of all 186 *L. monocytogenes* genomes used in this study.

Supplementary Table 3 SNP matrix obtained from SNP analysis.

Supplementary Table 4 Significant genes of the subpopulations “EpiCl + ReE” and “C2” revealed by genome wide association studies. In the columns are reported the variant (ID of the gene identified as significant), af (the proportion of samples where the variant is present in), filter-pvalue (association of the variant with the sub-population, unadjusted for population structure), lrt-pvalue (the *p*-value from the lmm association), beta (the effect of the variant), beta-std-err (the standard error of the fit on beta), variant_h2 (the variance in the sub-population explained by the variant) and product (product codified by the gene). The significant genes common between “EpiCl + ReE” and “C2” are highlighted in yellow.

Supplementary Table 5 Genomic characterization of the 186 *L. monocytogenes* genomes using BIGSdB Pasteur platform. Presence/absence of each genes was represented by 1 (presence), white cell (absence), I (incomplete allele).
